# The genetics of gaming: A longitudinal twin study

**DOI:** 10.1002/jcv2.12179

**Published:** 2023-05-28

**Authors:** Anders Nilsson, Ralf Kuja‐Halkola, Paul Lichtenstein, Henrik Larsson, Sebastian Lundström, Helena Fatouros‐Bergman, Nitya Jayaram‐Lindström, Yasmina Molero

**Affiliations:** ^1^ Department of Clinical Neuroscience Centre for Psychiatry Research Karolinska Institutet Stockholm Sweden; ^2^ Stockholm Health Care Services Stockholm Sweden; ^3^ Department of Medical Epidemiology and Biostatistics Karolinska Institutet Stockholm Sweden; ^4^ Gillberg Neuropsychiatry Centre Institute of Neuroscience and Physiology University of Gothenburg Gothenburg Sweden; ^5^ Center for Ethics, Law and Mental Health (CELAM) Institute of Neuroscience and Physiology University of Gothenburg Gothenburg Sweden

**Keywords:** adolescence, gaming, heritability, twin studies

## Abstract

**Background:**

Gaming is a popular past‐time activity among children and adolescents, but it there is also a possible link to negative consequences such as psychological distress and lowered academic achievement. However, there are fundamental knowledge gaps remaining regarding central characteristics of gaming such as heritability, stability over time, and sex differences. We examined the genetic and environmental contribution to gaming behavior, including sex differences, continuity and change, in a longitudinal cohort of twins.

**Methods:**

This is the first longitudinal twin study on gaming, involving 32,006 twins in Sweden. Parents were asked about the twins' gaming at ages 9, 15 and 18. We used univariate and multivariate twin analyses to estimate the relative contribution of genetic and environmental influences at each time‐point as well as across time. Sex‐differences were also explored.

**Results:**

The results showed large sex differences, where genetics explained more of the variance for boys (31.3%–62.5% depending on age) than for girls (19.4%–23.4%). Genetic factors explained an increasing amount of the variance for boys (31.3% at age 9, 62.5% at age 15 and 53.9% at age 18). Shared environmental factors explained a larger proportion of the variance among girls, which remained relatively stable over time (70.5% at age 9, 61.8% at age 15 and 60.5% at age 18). The results also indicated that most of the variance came from genetic and environmental sources specific to each age.

**Conclusions:**

Compared to many other behavioral phenotypes, such as gambling, gaming was relatively unstable with a large degree of genetic innovation. There were large sex differences in the contribution of genetic and environmental factors. This suggests that excessive gaming could be the result of age‐ and sex‐specific genetic and environmental factors, and should be taken into account when mapping gaming behaviors, since these behaviors might be under continual etiological transformation.

## Graphical abstract

Gaming is a popular past‐time activity but it might be linked to psychological distress and little is known about its heritability, stability over time and sex differences. This longitudinal study of 32,006 twins showed large sex differences where boys'; gaming was explained by genetics to a higher degree than girls'; gaming. The results also indicated that most of the variance came from genetic and environmental sources specific to each age.

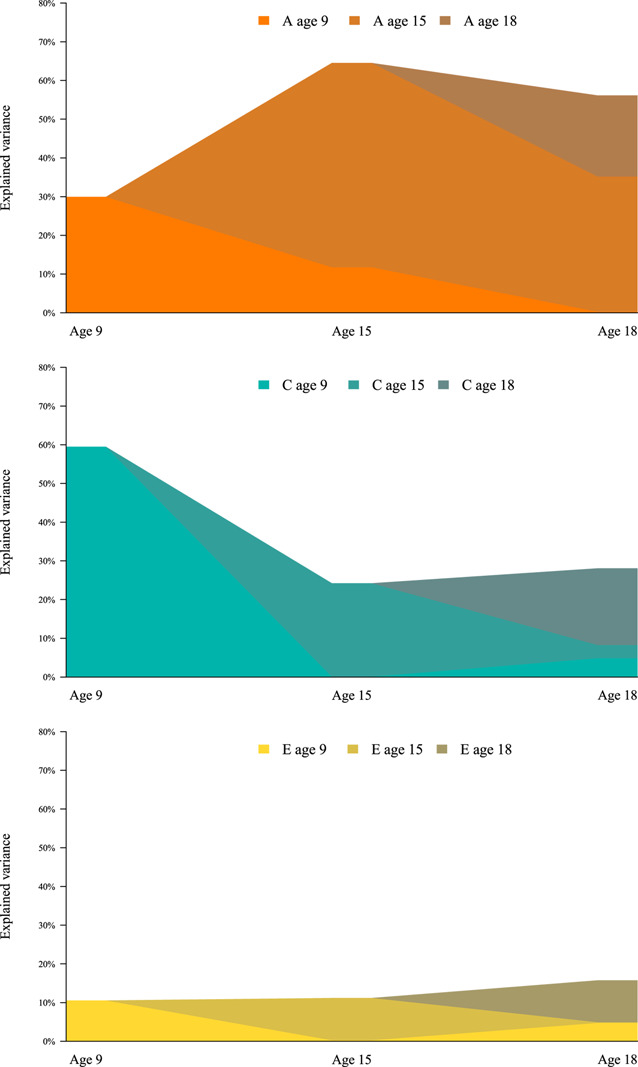

**Key points**

- Gaming is a popular past time activity, but excessive gaming has been linked to negative outcomes such as psychological distress.
- Little is known about the heritability of gaming.
- This study is the first ever longitudinal twin study on gaming, and also the first using a nation‐wide cohort.
- The study show that gaming heritability depends on age and sex, with particularly large differences between sexes.
- Gaming is less stable than many other phenotypes, which should be taken into account when mapping gaming behaviors among adolescents.

## INTRODUCTION

Gaming is a highly popular past‐time activities, with approximately 3 billion people playing video games worldwide an increase from 2 billion in 2015 (Wijman, 2020). In Europe, 60% of teenagers report gaming on school days, and 70% on non‐school days (Mokinaro et al., 2020). The average gamer have been reported to spend between 7 and 16 h a week on gaming (GamingSmart, 2020; The NPD Group, 2021), but certain subgroups tend to spend more than 4 h per day gaming (Office of Communications [Ofcom], 2022), making it one of the most popular spare time activities. Gaming is the act of playing video games, defined as *“a game which we play thanks to an audiovisual apparatus and which can be based on a story*” (Esposito, 2005), but games are heterogenous and vary in terms of genre, platform (e.g., computer, gaming console), number of players and objectives (King & Delfabbro, 2018). When gaming emerged in the 1970s and 80s, it was mostly based in arcades, while the 1990s saw a large‐scale introduction of games for home use. During the first decades of the 21st century, further technological development has enabled more advanced and interactive games and new modes of gaming through for example, smartphones or virtual reality equipment. This has generally made games more accessible and increasingly played online (King & Delfabbro, 2018). In the U.S. the percentage of the population playing a game (including video games, but also board games) on an average day doubled from 7.8% in 2003 to 14.0% in 2021, but the average time spent gaming has remained relatively stable (U.S. Bureau of Labor Statistics, 2022). However, excessive gaming has been linked to negative consequences such as psychological distress, sleep disturbances, neglected hygiene, and lower academic achievement (Brunborg et al., 2013, 2014; Carey et al., 2021), although the causality of such results largely remains obscured. Other studies have pointed to possible benefits of gaming, including enhanced problem solving, and social skills (Kovess‐Masfety et al., 2016; Reynaldo et al., 2021).

In the American Psychiatric Association's Diagnostic and Statistical Manual for Psychiatric Disorders (DSM‐5), *Internet gaming disorder* is suggested as a possible future diagnosis (Petry et al., 2015). In 2018, the World Health Organization decided to introduce *gaming disorder* in the 11th version of their International Classification of Diseases (ICD‐11). It is characterized by: (A) an impaired control over the gaming; (B) an increased priority given to gaming to the extent that it takes precedence over other interests and activities, and; (C) a continued involvement in gaming despite negative consequences (Billieux et al., 2021). However, the decision to include it in ICD‐11 caused some controversy (Aarseth et al., 2017; Van Rooij et al., 2018); critics pointed to a lack of research on the etiology of gaming disorder, and that the diagnosis could risk medicalizing a common and generally harmless activity (Van Rooij et al., 2018).

There is a relative consensus that excessive gaming can be associated with significant harm (Van Rooij et al., 2018), although proposed explanations vary greatly; including features of the games themselves (James & Tunney, 2017; King et al., 2011), personality traits (Braun et al., 2016; Dieris‐Hirche et al., 2020; Şalvarlı & Griffiths, 2022), biological vulnerabilities such as impairments in inhibition (Argyriou et al., 2017), and gaming in order to regulate emotions (Marchica et al., 2019). It has been proposed that excessive gaming could be one of several behavioral addictions (e.g., gambling disorder, compulsive shopping), with similar psychological and biological underpinnings, such as deficits in decision making, cue reactivity and imbalances in the fronto‐striatal circuits (connectivity between amygdala and medial prefrontal cortex, responsible for higher order emotional and cognitive functioning) (Brand et al., 2019; Clark, 2014).

Despite the number of gamers being counted in billions, and despite accompanying concerns on possible negative effects, there is a lack of research on the fundamental features of gaming behavior, including heritability, stability and change, and sex differences. Longitudinal twin studies are well suited to address these types of questions, since it offers the possibility to estimate genetic and environmental influences on specific traits and behaviors. This is essential to understand gaming itself, when there is a risk to develop maladaptive forms of gaming, how it should be reliably assessed and could support the development of prevention and intervention measures. Only a few studies have investigated gaming using twin methodology: in a study of 2635 16 year old UK twin pairs, genetics accounted for 39% of the time spent on online gaming (Ayorech et al., 2017). Of the different types of online media use studied (e.g., Facebook use, visiting entertainment websites), gaming was the only studied behavior without gender differences in its heritability. In a Dutch study of “compulsive Internet use” in 5247 twin pairs, including online gaming, 48% of the variance was explained by genetic factors, with no significant differences between sexes (Vink et al., 2016). The lack of sex differences in heritability is worth noting in light of the sex differences in time spent gaming, where boys are estimated to spend twice as much time gaming as girls (Mokinaro et al., 2020; Veltri et al., 2014). However sex differences in heritability may change over time (Plomin & Deary, 2015). Since these studies were cross‐sectional in nature, they were unable to distinguish between stable genetic and environmental factors, and factors that emerge during development. Consequently, there is little knowledge about the dynamic nature of the genetic factors for gaming. A better understanding of this would clarify the phenotypic characteristics of gaming, and could inform theories about gene‐environmental processes in gaming, such as *genetic amplification* (i.e. genetic differences are amplified as individuals select, modify, and create environments that are correlated with their genetic propensities) and *innovation* (i.e., explaining phenotypes by novel genetic influences not present at earlier ages) (Plomin & Deary, 2015). This knowledge could have implications for how we understand gaming behaviors, the conceptualization of gaming disorder, and for its treatment and prevention.

## RESEARCH QUESTIONS

This study examined continuity and change in genetic and environmental effects for gaming in a cohort of 32,006 twins who were followed up at ages 9, 15 and 18. By applying a longitudinal design, we could discriminate between developmentally stable factors (e.g., genetic factors that predict that the genetic liability originates solely from a single set of genes that influence gaming), and developmentally dynamic factors (e.g., new genetic and environmental effects that emerge over development).

We examined four research questions:

1. What is the relative genetic and environmental contribution to gaming behavior?
2. Are there sex differences in the relative genetic and environmental contribution to gaming behavior?
3. Is gaming frequency in childhood (9 years) associated with gaming behavior in adolescence (15 and 18 years)?
4. What is the relative genetic and environmental contribution for continuity and change in gaming behavior?

## MATERIALS AND METHODS

### Sample

The data is from the ongoing Child and Adolescent Twin Study in Sweden (CATSS) (Anckarsäter et al., 2011). In brief, parents to all twins born in Sweden since 1992 were contacted around their ninth birthday and asked to participate in a telephone interview. The questions on gaming were incorporated in the telephone interviews in 2007, which means that not all participants were asked about gaming at age 9 (i.e., questions were asked only to those born from July 1998 and onwards at the age 9 wave). The data was updated until 2020. The parents were asked to answer an online questionnaire when the twins were 15 (from 2010) and 18 (from 2011) years old. General response rates for the CATSS study has been described elsewhere (Zagai et al., 2019). At the current data extraction, parents of 37,055 twins had participated in CATSS on at least one occasion. Out of these, 4525 (12.2%) participated in the age 9 questionnaire before the gaming question was introduced, but chose not to participate in age 15 or age 18 questionnaires. Thus, in the current study, parents had participated in questionnaires regarding 32,530 twins (87.8% of the original sample). For 32,006 of these twins (98.4% of those participating), their parents had provided data about gaming on at least one occasion. Henceforth these 32,006 twins are referred to as the “analytic sample.” At age 9, a total of 21,891 out of 21,925 (99.8%) who were asked about gaming provided data, at age 15 it was provided for 13,402 out of 14,157 (94.7%), and at age 18 data was provided for 9767 out of 10,315 (94.7%).

### Measures

#### Gaming variable

At each occasion, parents were asked “how often does your child engage in the following spare time activity—computer gaming—for at least half an hour per time?” and “how often does your child engage in the following spare time activity—tv gaming—for at least half an hour per time’.” Answers were ranked on an ordinal scale: “never,” “once a month,” “1–2 times per week,” “3–6 times per week,” and “almost daily.” We chose to combine the questions on computer gaming and tv gaming into one variable, where we identified the highest gaming frequency in either of the two questions and ignored the other, potentially lower value. If the answer to one question (e.g., computer gaming) was missing, we chose the other answer (e.g., tv gaming). These variables are henceforth referred to as “gaming questions.”

#### Covariates

We used recorded biological sex as a stratification in our analyses. We used birth year for adjustment, to account for potential confounding effects of changing gaming behavior over calendar time, for example, cohort effects in terms of time spent gaming due to changes in types of games played and how they are played.

### Analyses

#### Descriptive

We summarized the absolute number and proportions of individuals in the sample by sex, birth year categories (1992–1995, 1996–2000, 2001–2005, 2006–2010), answered gaming questions (i.e., non‐missing response) at the different ages (9, 15, 18, and all three ages combined), and zygosity. We calculated these numbers for the full sample, and separately by each age when the gaming questions were asked. Further, we calculated the absolute numbers and proportions of the different gaming frequency categories separately by boys and girls.

#### Age‐specific analyses

We estimated polychoric correlations between the reported gaming frequencies between twins in pairs, within each age separately; we refer to these correlations as intraclass correlations (ICC). Polychoric correlations are measures of association between two ordered categorical variables. Shortly, they are the correlations between two normal distributions, which we assume underlie the ordered categorical variables observed (e.g., our measure of gaming frequency). For estimation, the assumption is that there are thresholds, estimable from data, which separate the categories (4 thresholds for the current five categories of gaming frequency). Everyone is assumed to have a value on the continuous underlying scale, but we imperfectly observe this value by which category they belong to depending on between which thresholds this value lies. The polychoric correlation is the correlation between the assumed underlying continuous normal distributions, which is inferred through the observed pattern of association between the categorized version we have observed (Ekström, 2011).

We estimated ICCs separately by sex‐zygosity combinations, that is, monozygotic (MZ) females, MZ males, dizygotic (DZ) females, DZ males, and DZ opposite sexed twin pairs. We estimated these in a structural equation framework, both unadjusted and adjusted for birth year. If MZ twins have a higher ICC than DZ twins, then, under the assumption of equal environmental contributions to similarity of phenotype between MZ and DZ twins (Plomin et al., 2008), we infer that genetics has an impact on the phenotype under study. In addition, because MZ twins share essentially 100% of genetics, while DZ twins share 50% of their co‐segregating alleles, if the association in DZ twins is greater than half of that in MZ twins, we also infer contributions of shared environment to the phenotypic variation. The amount to which MZ twins, who share genetics and environment, have a correlation less than 1 is attributed to individually unique, or non‐shared, environmental contributions to the phenotypic variation. We performed post‐hoc tests of whether the difference between MZ and DZ twins differed between same‐sexed males and females, separately for the three ages, using Wald type tests in combination with the delta method.

We then proceeded to estimate the contributions from additive genetics (A; the heritability), shared environment (C), and non‐shared environment (E; which also includes measurement error) to the variance in gaming frequency at ages 9, 15, and 18; we refer to this model as the ACE‐model. The model used classic twin methodology, reliant on the assumptions about genetic and environmental sharing outlined above, and was fitted as structural equation models. In the model, A‐contributions to variance were assumed to be perfectly correlated between twins in MZ pairs, and correlated 0.5 in DZ pairs. Contributions from C were assumed to correlate 1.0 in both MZ and DZ pairs. Contributions from E was assumed to be individual‐specific, that is, correlated 0 between twins. Since the distribution of gaming time varied extensively between boys and girls, and because associations between DZ twins was quite different between sexes and particularly among opposite sexed DZ twins, we fitted so‐called sex‐limitation models. In these models we estimated separately the A, C, and E contributions to variance in boys and girls; differences in magnitude of these are referred to as quantitative sex‐differences. We also estimated a genetic correlation between A‐contributions in boys and girls, referred to as qualitative sex‐differences. This was achieved by attributing lower correlations in opposite sexed twins, compared to expected correlation based on same‐sex twins, to a less than expected correlation between A in boys and girls. We performed post‐hoc tests of whether A, C, and E contributions to variance differed between males and females, separately for the three ages, using Wald type tests in combination with the delta method.

#### Longitudinal analysis

We analyzed the phenotypic stability of gaming, that is, the association between gaming frequency reported over age, using polychoric correlations. First, we estimated polychoric correlations between gaming frequency age 9 and age 15, between age 9 and age 18, and between age 15 and 18, both unadjusted and adjusted for birth year, within a structural equation framework, separately for boys and girls. For these analyses, only those who responded at both time points were included, thus different number of individuals were included for different analyses. Then, we fitted a trivariate quantitative genetic model, based on the same assumptions as for the univariate ACE‐models above. In this model, not only the variances of gaming within each age were modeled to be due to A, C, or E, but also the association between the measures at each age were modeled by these sources. Specifically, we modeled the variance at each age to be due to A, C, and E sources originating from the current age or from the preceding age(s); age 9 had variance contributions from A, C, and E originating at age 9; age 15 from age 9 and age 15; and age 18 from age 9, age 15, and age 18. This model is often referred to as the “Cholesky model,” and is suitable to investigate development over time and the relative genetic and environmental contribution to variance. We fitted the model unadjusted for covariates, because the covariates had shown little‐to‐none effect on the univariate estimates, and in boys and girls separately since we identified sex differences in univariate ACE‐models. We excluded the opposite sexed twin pairs because the qualitative sex differences were not appropriate to model with standard twin methodology—the trivariate analysis was the only analysis where we excluded opposite sexed twins.

### Sensitivity analyses

As sensitivity analyses, we investigated the ICCs and univariate ACE‐analyses adjusted for birth year. We performed this adjustment by allowing the mean of the assumed underlying normally distributed liability to vary according to its regression on birth year (linear and quadratic effects), while keeping the thresholds fixed.

For all analyses we used the statistical software R version 4.0.5, for estimation of polychoric correlations and ACE‐models (univariate and trivariate) we used library OpenMx version 2.19.6 (Neale et al., 2016). Model fitting was performed using Full Information Maximum Likelihood, which makes use of all available data and handles missing data appropriately (Newman, 2003).

## RESULTS

### Descriptive

The descriptive statistics outlined in Table 1 show the characteristics of the included participants, with distribution of zygosity, sex, and response rates. The analytic sample consisted of 32,006 twins, of which 49.7% were males, 30.5% were MZ twins, 34.1% were DZ same sexed twins, and 33.9% were DZ twins of opposite sexes.

**TABLE 1:** Descriptive.

	Boys	Girls
Total^a^ (row percent)	15,903 (49.7%)	16,103 (50.3%)
Birth year		
1992–1995	2429 (15.3%)	2584 (16.0%)
1996–2000	4486 (28.2%)	4576 (28.4%)
2001–2005	4903 (30.8%)	4836 (30.0%)
2006–2010	4085 (25.7%)	4107 (25.5%)
Observed gaming measure,^b^ (%) of eligible sample		
Age 9	10,987 (99.9%)	10,904 (99.9%)
Age 15	6464 (96.9%)	6938 (96.8%)
Age 18	4697 (96.7%)	5070 (96.2%)
Age 9, 15, and 18	1043 (92.6%)	1153 (91.0%)
Zygosity		
MZ	4594 (28.9%)	5158 (32.0%)
DZ same sexed	5630 (35.4%)	5300 (32.9%)
DZ opposite sexed	5426 (34.1%)	5420 (33.7%)
Unknown	253 (1.6%)	225 (1.4%)

*Note*: Number of individuals (column percent), unless otherwise specified.

Abbreviations: DZ, dizygotic; MZ, monozygotic.

^a^ Refers to the analytical sample of 32,006 twins.

^b^ Having a non‐missing value for the gaming measures at the different ages.

**FIGURE 1:**
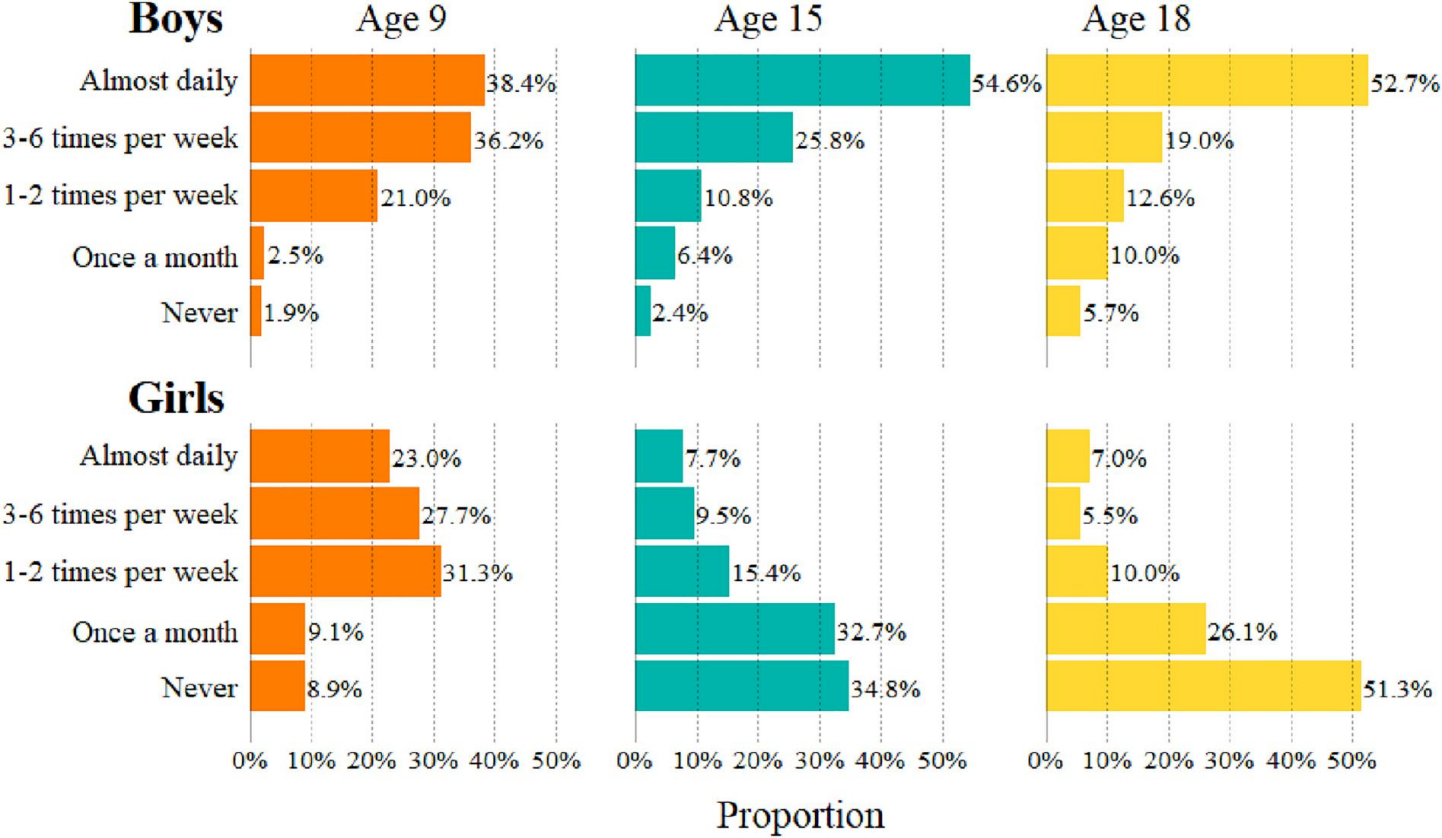
Gaming frequency by age and gender.

Figure 1 (with absolute number of individuals in Supporting Information S1: Table S1) shows gaming frequency for each of the three waves, divided by sex. In general, boys and girls displayed different patterns, where a greater proportion of boys gamed almost daily at age 15 (54.6%) and age 18 (52.7%) as compared to at age 9 (38.4%). However, parents also reported that a greater proportion of boys never gamed at age 18 (5.7% as compared to 1.9% at age 9). Girls showed a different development, where 23.0% of age 9 gamed almost daily, compared to 7.5% and 7.0% at ages 15 and 18, respectively. The proportion of girls who never gamed increased from 8.9% at age 9%–51.3% at age 18. Taken together, boys tended to spend more time gaming at ages 15 and 18, while girls tended to spend less time gaming at age 15 compared to 9, and even less at age 18 compared to age 15.

### Age‐specific univariate analyses

The ICCs of the different types of twin pairs' gaming frequency at each age wave are presented in Table 2. Correlations were higher at age 9 for all groups, with gradually lower correlations for each consecutive age wave. Across age waves, MZ twins showed higher ICCs than DZ twins, with greater differences for males compared to females (*p*‐values 4.8 × 10^−4^, 6.1 × 10^−10^, and 2.5 × 10^−4^ for age 9, 15, and 18, respectively). The lowest correlations across age groups were found among DZ twins of different sexes.

**TABLE 2:** Observed ICC by sex, zygosity and age (95% CI).

	Age 9	Age 15	Age 18
MZ female	0.90 (0.89–0.91)	0.85 (0.83–0.87)	0.85 (0.82–0.87)
MZ male	0.89 (0.88–0.90)	0.89 (0.88–0.91)	0.84 (0.81–0.87)
DZ female	0.80 (0.79–0.82)	0.73 (0.70–0.76)	0.73 (0.68–0.77)
DZ male	0.73 (0.71–0.76)	0.58 (0.53–0.63)	0.57 (0.51–0.63)
DZ opposite sex	0.54 (0.51–0.57)	0.26 (0.21–0.31)	0.28 (0.21–0.34)
*p*‐value difference between sexes^a^	4.8 × 10^−4^	6.1 × 10^−10^	2.5 × 10^−4^

Abbreviations: DZ, dizygotic; ICC, intraclass correlations; MZ, monozygotic.

^a^ Test whether the difference between MZ and DZ is greater among same sexed males than among same sexed females; that is, a test of the contrast “(MZ male—DZ male)—(MZ female—DZ female).” Wald type test using delta method.

Results from univariate heritability analyses are presented in Table 3, showing the contributions of A, C and E at each age wave. The genetic contribution was higher for males than females at all time points (*p*‐values <.001), and almost doubled for males between age 9 and 15 (from 31.3% to 62.5%), while only increasing slightly for females (from 19.4% to 23.4%). The contribution of shared environment was substantially higher for females throughout ages (*p*‐values <.001), and higher at age 9 compared to the following time points for both sexes. Non‐shared environment was similar for both sexes and increased with age (*p*‐values for difference across sexes were 0.14, 6.3 × 10^−4^, and 0.74 for age 9, 15, and 18, respectively).

**TABLE 3:** Estimated univariate contribution to gaming from genetics (A), shared environment (C) and non‐shared environment (E) (95% CI).

	Age 9	Age 15	Age 18
A female	19.6% (15.7%–23.6%)	23.4% (16.7%–30.1%)	24.3% (15.1%–33.6%)
A male	31.3% (26.1%–36.5%)	62.5% (52.1%–73.0%)	53.9% (41.1%–66.7%)
C female	70.5% (66.9%–74.2%)	61.8% (55.7%–67.8%)	60.5% (52.0%–68.9%)
C male	57.7% (52.8%–62.5%)	26.9% (16.8%–37.1%)	30.3% (18.3%–42.2%)
E female	9.8% (8.8%–10.9%)	14.9% (13.0%–16.7%)	15.2% (12.8%–17.7%)
E male	11.1% (9.8%–12.3%)	10.5% (8.8%–12.2%)	15.9% (12.9%–18.8%)
r_fm	−0.810 (−1.217 to −0.404)	−0.794 (−1.370 to −0.217)	−0.839 (−1.568 to −0.109)
*p*‐value sex‐difference A^a^	4.7 × 10^−4^	6.2 × 10^−10^	2.5 × 10^−4^
*p*‐value sex‐difference C^a^	3.2 × 10^−5^	8.9 × 10^−9^	5.4 × 10^−5^
*p*‐value sex‐difference E^a^	0.14	6.3 × 10^−4^	0.74

*Note*: “r_fm” are estimated genetic correlations between males and females. Negative values are not readily interpretable.

^a^ Test between A, C, and E contribution to variance among males and females. Wald type test using delta method.

### Longitudinal analyses

Table 4 shows the stability of gaming across all time points divided by sex (with figures adjusted for birth year in Supporting Information S1: Table S2). Results show an increase in the stability in gaming at older ages for both males and females, meaning that the correlations between age 15 and 18 were higher than those between age 9 and age 15 and age 18. In sensitivity analyses we further adjusted for birth year (Supporting Information S1: Table S2), and results remained very similar.

**TABLE 4:** Stability of gaming over age as polychoric correlations (95% CI).

			Polychoric correlations	
			Age 15	Age 18
Boys	Age 9	Estimate	0.26 (0.21–0.31)	0.25 (0.19–0.32)
		Sample size^a^	2896	1352
	Age 15	Estimate	‐	0.56 (0.52–0.59)
		Sample size^a^	‐	3040
Girls	Age 9	Estimate	0.20 (0.16–0.25)	0.22 (0.15–0.29)
		Sample size^a^	3194	1449
	Age 15	Estimate	‐	0.51 (0.48–0.54)
		Sample size^a^	‐	3319

^a^ Sample size with non‐missing values at both ages.

Figure 2A,B show the explained variance over time from A, C and E, for males and females, respectively (Table 5 includes the same information with 95% confidence intervals). The figures display the contribution of A, C and E for each age wave, and their contribution to the explained variance for the following age waves, that is, the stability and innovation of what explains gaming behavior. Most of the variance at each time point reflected variance specific to that age. For males at age 15, 21.2% of the variance came from age 9 genetics, but the rest of the ACE variance from age 15. At age 18, the genetic contribution for males came from all three ages (2.3%, 15.2% and 37.3% respectively), while the shared environment contribution only came from earlier time points (4.6% from age 9% and 24.8% from age 15).

FIGURE 2(A) Explained variance over time from genetics (A), shared environment (C), and non‐shared environment (E), males. The figure displays the contribution to the variance from genetics, shared environment and non‐shared environment. It also displays from which age the variance originates from. Variance from the later ages is illustrated with darker colors. (B) Explained variance over time from genetics (A), shared environment (C), and non‐shared environment (E), females. The figure displays the contribution to the variance from genetics, shared environment and non‐shared environment, and from which age the variance originates from. Variance from the later ages is illustrated with darker colors.

**Figure:**
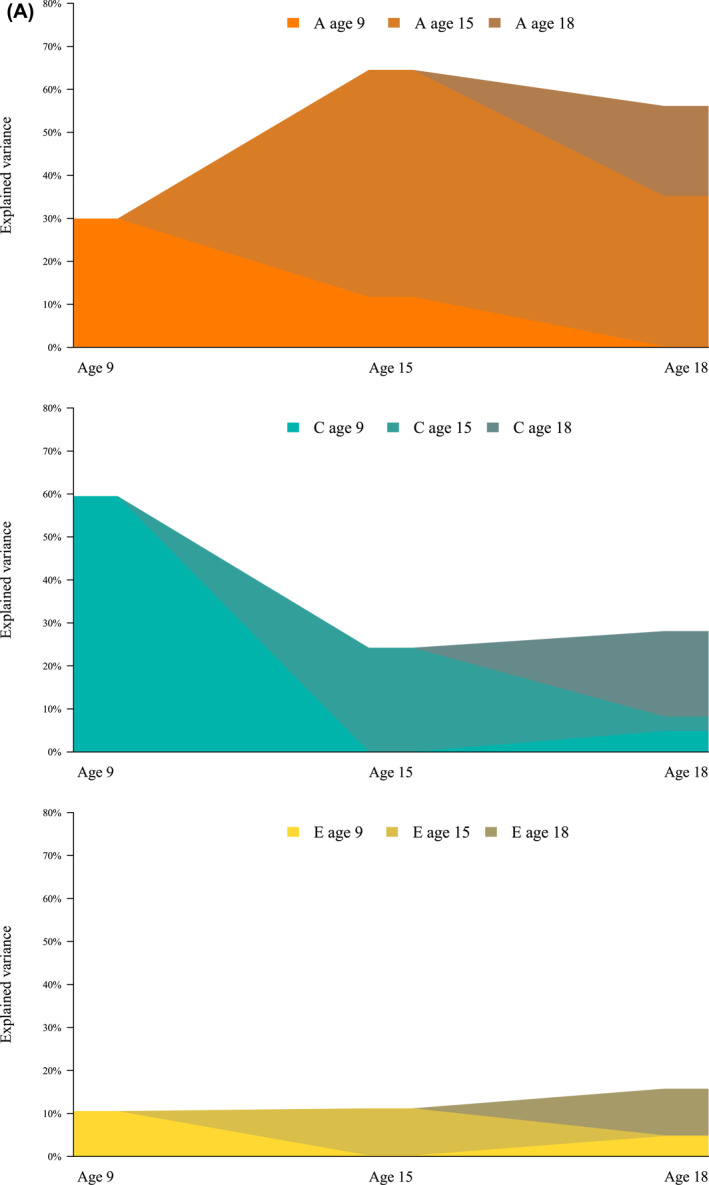

**Figure:**
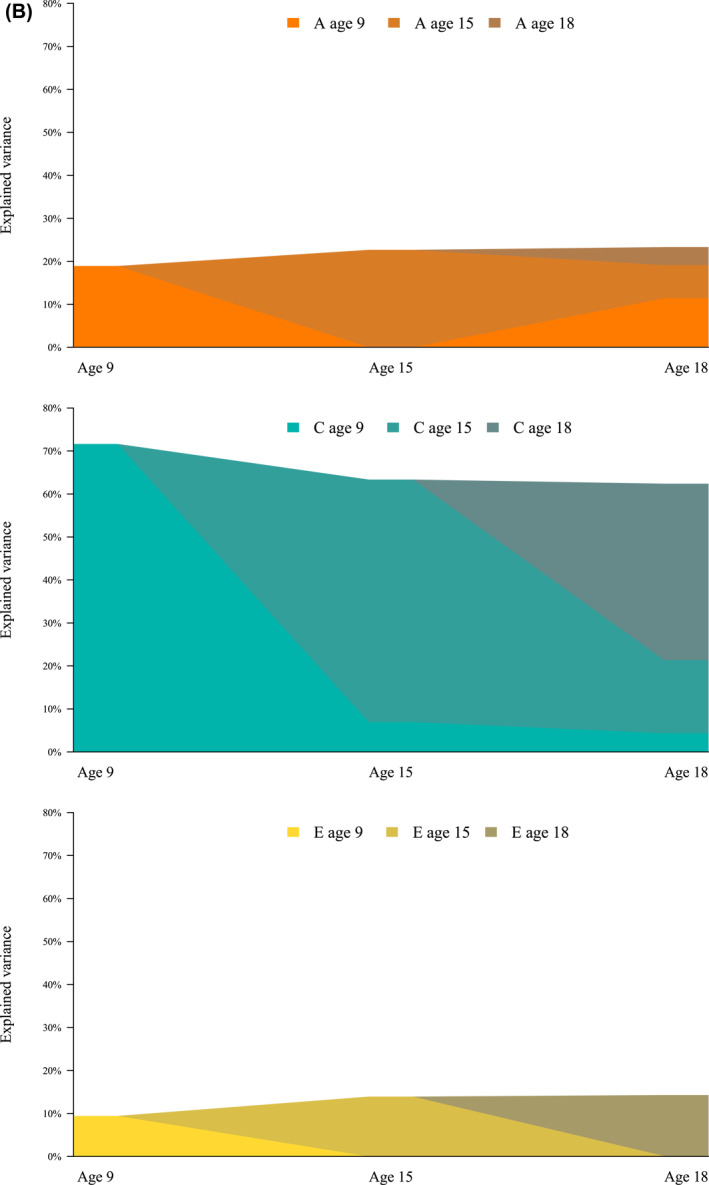

**TABLE 5:** Explained variance from additive genetics (A), shared environment (C), and non‐shared environment (E), Estimates (95% confidence intervals) are presented in percent.

		From age 9	From age 15	From age 18	Total
Females					
To age 9	A	18.9 (14.7–23.2)	NA	NA	18.9 (14.7–23.2)
	C	71.6 (67.7–75.6)	NA	NA	71.6 (67.7–75.6)
	E	9.4 (8.4–10.5)	NA	NA	9.4 (8.4–10.5)
To age 15	A	0.0 (−0.1–0.1)	22.7 (16.7–28.7)	NA	22.7 (16.7–28.7)
	C	6.9 (2.7–11.1)	56.4 (49.7–63.1)	NA	63.4 (57.8–68.9)
	E	0.0 (−0.0–0.0)	13.9 (12.1–15.7)	NA	13.9 (12.1–15.7)
To age 18	A	11.4 (2.1–20.8)	7.7 (1.2–14.3)	4.2 (−3.7–12.0)	23.3 (14.1–32.5)
	C	4.4 (−2.7–11.5)	17.0 (5.2–28.8)	41.0 (32.9–49.2)	62.4 (53.8–71.0)
	E	0.0 (−0.1–0.1)	0.1 (−0.2–0.4)	14.2 (11.7–16.6)	14.3 (11.8–16.8)
Males					
To age 9	A	29.9 (24.0–35.8)	NA	NA	29.9 (24.0–35.8)
	C	59.5 (54.0–65.0)	NA	NA	59.5 (54.0–65.0)
	E	10.6 (9.3–11.8)	NA	NA	10.6 (9.3–11.8)
To age 15	A	11.7 (5.8–17.7)	52.8 (44.1–61.5)	NA	64.5 (56.0–73.1)
	C	0.1 (−0.3–0.5)	24.2 (16.0–32.4)	NA	24.2 (16.0–32.5)
	E	0.2 (−0.3–0.8)	11.0 (9.1–12.9)	NA	11.2 (9.4–13.1)
To age 18	A	0.2 (−0.6–1.0)	34.9 (28.9–41.0)	21.0 (17.1–24.8)	56.1 (49.3–63.0)
	C	4.9 (NA‐NA)^a^	3.4 (1.3–5.5)	19.8 (16.0–23.6)	28.1 (21.8–34.4)
	E	4.8 (1.3–8.3)	0.2 (−0.4–0.7)	10.8 (6.8–14.8)	15.8 (12.8–18.7)

*Note*: A graphic summary is also shown in Figure 2A,B. Model was fitted using Full Information Maximum Likelihood. Confidence intervals are Wald‐type, hence they can span outside parameter bounds (lower bound lower than 0 for variance contributions).

Abbreviation: NA, not applicable.

^a^ Due to instability in model optimization we could not obtain standard errors.

At age 15, all variance for females consisted of ACE variance from age 15, except for a 6.1% shared environment contribution from age 9. At age 18, all of the genetic contribution for females (25.5% of the total variance) came from earlier ages, while most of the contribution for the shared environment came from age 18 (4.1% of the total variance from age 9, 13.7% from age 15% and 41.3% from age 18). Virtually all of the non‐shared variance for both females and males at age 18 came from age 18.

### Sensitivity analyses

In our sensitivity analyses, we examined if adjustment for birth year changed ICCs and univariate ACE results (Supporting Information S1: Tables S2–4). Results showed no or very small differences compared to the unadjusted figures.

## DISCUSSION

This study investigated the relative contribution of genetic and environmental factors, sex differences, stability, and change of gaming in a cohort of 32,006 twins. To the best of our knowledge, this is the first study to use a longitudinal twin design to analyze genetic and environmental contribution to gaming behavior, as well as sex differences. This is also the first genetic study to analyze gaming behavior, rather than problematic gaming behaviors. In brief, our results showed that genetics explained 19%–63% of the gaming behavior, depending on age and sex. The genetic influence increased with age for boys, but remained stable for girls. There were substantial differences between male and female gaming, where girls' gaming was explained by shared environmental factors to a higher degree. Gaming was relatively unstable for both boys and girls, but became more stable with older ages. The results show no substantial changes when adjusting for year of birth. This indicates a lack of cohort effects, such as changes in how games are played, that have impacted the results. These results provide novel and unique insights into the longitudinal phenotypic expression of gaming.

Our study showed a heritability of 31%–63% for boys, and 19%–23% for girls (depending on age). Our heritability estimates are similar to those reported by Ayorech et al. (2017), where the genetic contribution among 16‐year‐olds was 39%. However, while they found no sex differences, we showed that genetics explained a larger degree of gaming for boys than for girls. This is likely due to our study's ability to capture sex differences at different time points, which provides a better overall account. The influence of genetics increased over time for boys. One potential explanation to this finding is active gene‐environment correlation, that is, *genetic amplification*, where small genetic differences are amplified as children grow older and to a greater extent select, modify, and create environments that are correlated with their genetic propensities compared to when they are younger. Similar findings have been observed for intelligence, in which the genetic contribution increases from around 20% in infancy to 80% in adulthood (Plomin & Deary, 2015). Active gene‐environmental correlation processes may partly offer an explanation on how the genetic propensity to gaming behavior becomes amplified with age as individuals select and create environments for gaming, at least for boys. Girls, on the other hand, might be less encouraged to game than boys, which has been proposed by previous research (Lopez‐Fernandez et al., 2019). Girls tend to game less than boys, which previous research has suggested could be due to a combination of a gaming culture that mainly caters to males, sexist portrayals of female game characters, sexual harassment of female players and negative expectations based on gender (Lopez‐Fernandez et al., 2019). This could also result in sex differences in the phenotypical expression of gaming. Similar patterns have been found for “problematic internet use,” prompting the authors to suggest that social norms restrict girls' genetic propensities to expression (Li et al., 2014). Girls also tend to spend more time than boys using social media (Mokinaro et al., 2020), which could possibly be a different expression of the same genetic propensity.

In the present study, shared environment explained a substantial part of gaming across age and sex. For both girls and boys at age 9, the results from our study contradict one of the “laws of behavior genetics,” which state that the effect of being raised in the same family is smaller than the effect of genes (Turkheimer, 2000). In our study, the influence of shared environment was higher than in other phenotypes (e.g. attention deficit hyperactivity disorder, learning difficulties, and eating disorders) studied in the same cohort (Anckarsäter et al., 2011). However, these studies mainly focused on disorders rather than behaviors. A more relevant comparison could be gambling, since both behaviors share many features, such as the structural characteristics of the games and possible common pathways in developing problematic behaviors (Clark, 2014). In a meta‐analysis of twin studies, genetics explained on average 28% of non‐problematic gambling among females, and 47% among males, while non‐shared environment explained more than 50% for both sexes. Shared environment explained only a small amount of the variance among the females (14%), and none among males (Xuan et al., 2017). These results are in keeping with our results regarding the genetic contribution, but differ substantially in terms of the environmental contribution. In our study, the contribution of non‐shared environment was much smaller than the contribution of shared environment. The differing results could be due to age differences in phenotypical expression; gambling is predominantly an adult activity, and thus more likely to be influenced by non‐shared environment. In contrast, gaming is likely influenced by family factors such as parental supervision and access to video games or computers. This is supported by the fact that the contribution of shared environment diminished sharply for boys at older ages, although it remained stable for girls. This could be explained by decreased parental control over time, enabling a fuller expression of a genetic propensity among boys to play video games. The high degree of shared environment contribution could also be partly due to a rater bias where parents would tend to assign similar responses for both twins, thus inflating the shared environments contribution to variance. However, the opposite phenomenon, where parents tent to overemphasize differences, called “the sibling contrast effect” has been observed in previous studies (Simonoff et al., 1998). This would suggest that the high degree of shared environment contribution represents a correct estimate of the explained variance for gaming.

The longitudinal analyses indicated that gaming frequency at age 9 to some extent predicted gaming frequency at later ages, but with low stability compared to phenotypes such as gambling (King et al., 2017) and physical activity (Aarnio et al., 2002). Previous research focused on the temporal stability of problematic gaming (with follow‐up measures after 1–3 years) has yielded heterogenous results; spanning between 20% and 84% (Richard et al., 2020), and with greater stability among adolescents than among adults. It is likely that gaming is often cyclic in nature, where periods of more intense gaming alternate with less frequent gaming. The longitudinal Cholesky analyses mirrored these results by showing that most of the variance in gaming frequency was explained by sources originating from the current age. This is indicative of a process of *innovation*, where a phenotype can be explained by novel genetic influences that were not present at previous ages. Innovation might arise from genetic influences such as hormonal changes associated with puberty, or environmental changes such as a transition from primary school to middle school. In general, innovation is considered to be more prevalent at younger ages, whereas amplification becomes more important with age (Briley & Tucker‐Drob, 2013). The findings from this study corroborates this. Genetic variance for females at age 18 and shared environment variance for males at age 18 were mostly explained by sources origination from previous ages, while the source of variance for all the other measurement points was largely age‐specific. In conclusion, gaming seems to be a rather unstable activity with mostly novel genetic and environmental influences at each age. This could have important implications for the understanding of gaming as a possibly problematic behavior, indicating that problematic gaming patterns might be the result of age‐specific genetic and environmental factors. For example, in a clinical setting, these findings (i.e. instability and genetic innovation) should be taken into account when mapping gaming behaviors among adolescents, since these behaviors might be under ongoing transformation.

The results from this study provide a more in‐depth understanding of the genetic and environmental influences of gaming, stability and sex differences. Gaming is constantly changing, where some games and platforms become obsolete and are replaced by novel ways of gaming. More comprehensive conceptualizations of gaming, irrespectively of its current mode of expression, could possibly capture other aspects regarding generational or sex differences. Future research should also investigate whether the genetic and environmental contribution overlaps with other phenotypes, such as impulsivity, since it has been identified as an important factor in the development of gaming disorder (Şalvarlı & Griffiths, 2022). Furthermore, there is a need to study similar activities, such as internet use or social media activity, since previous research has suggested substantial overlap (Brand et al., 2019; Clark, 2014), and also to examine whether girls pursue these activities to a greater extent than gaming, as has been suggested in some studies (Mokinaro et al., 2020; Nilsson et al., 2022).

## STRENGTHS AND LIMITATIONS

To our knowledge, this is the first longitudinal cohort twin study assessing gaming behavior. It is also the first to use a population‐wide cohort, and assessing three age waves. That said, there are limits to bear in mind. First of all, not all participants have provided data on all time points, partly because the data collection for CATSS is still ongoing. However, by using Full Information Maximum Likelihood, we made use of all available data. Second, data is based on parental reports, and parents' awareness of their children's gaming could be limited, especially for adolescents. They might also have difficulties distinguishing between the gaming activities of two twins. Third, questions on gaming included only games played on computer or gaming consoles, and excluded games that can be played on telephones or tablets. The questions are also limited to estimating the frequency of video and/or computer gaming, whereas measures such as time spent gaming measured in hours and minutes, type of game played, motives to play or whether gaming is usually pursued alone or with others that could have provided more detailed information is not included. Fourth, there are 6 years between the first and second measuring points, but only 3 years between the second and third. Another measuring point between the first and second measurement could have provided more precise information on gaming during an often important transitional period in the lives of children and adolescents, in particular since our results indicated that gaming was the subject of a high degree of genetic innovation. Also, opposite sex twins were excluded from the trivariate analyses, due to the large phenotypical differences between sexes. Accordingly, we were unable to make use of all available data for the trivariate quantitative genetic model analyses. Furthermore, gaming might have changed during the time data was collected in terms of what games are played and how they are played. There is also a risk of systematic errors. However, the high heritability and relatively low unique environment, as well as the stability between ages 15–18, suggests this was not the case.

## CONCLUSIONS

This study is the first longitudinal twin study on gaming frequency. In brief, our results showed that genetics explained 19%–63% of gaming frequency, depending on age and sex. The genetic influence increased with age for boys, but remained stable for girls. There were substantial differences between male and female gaming patterns, where girls' gaming was explained by shared environmental factors to a higher degree. The results also showed that gaming was unstable compared over time, with a large degree of genetic innovation. This suggests that the developmental pathway of gaming is influenced by both instability and genetic innovation, and that this should be taken into account when mapping gaming behaviors among adolescents.

## AUTHOR CONTRIBUTIONS

**Anders Nilsson**: Conceptualization; investigation; methodology; project administration; writing – original draft; writing – review & editing. **Ralf Kuja‐Halkola**: Conceptualization; data curation; formal analysis; methodology; software; visualization; writing – review & editing. **Paul Lichtenstein**: Resources; supervision; writing – review & editing. **Henrik Larsson**: Resources; supervision; writing – review & editing. **Sebastian Lundström**: Supervision; writing – review & editing. **Helena Fatouros‐Bergman**: Conceptualization; supervision; writing – review & editing. **Nitya Jayaram‐Lindström**: Conceptualization; funding acquisition; supervision; writing – review & editing. **Yasmina Molero**: Conceptualization; data curation; formal analysis; investigation; methodology; project administration; supervision; writing – original draft; writing – review & editing.

## CONFLICT OF INTEREST STATEMENT

HL reports receiving grants from Shire Pharmaceuticals; personal fees from and serving as a speaker for Medice, Shire/Takeda Pharmaceuticals and Evolan Pharma AB; and sponsorship for a conference on attention‐deficit/hyperactivity disorder from Shire/Takeda Pharmaceuticals and Evolan Pharma AB, all outside the submitted work. He is also the Editor‐in‐chief of Journal of Child Psychology and Psychiatry Advances. PL serves on the JCPP Advances Editorial Advisory Board.

## ETHICAL CONSIDERATIONS

The participants in the CATSS study are protected by the informed consent, in which they are informed what is being collected and given the option to withdraw their consent and discontinue their participation. The study was approved by the Regional Ethical Review Board of Stockholm with registration numbers 02‐289; 09‐739; 10‐322; 10‐597; 10‐1410; 15‐1947.

## Supporting information

Supporting Information S1

## Data Availability

Data may be obtained from a third party and are not publicly available. The Public Access to Information and Secrecy Act in Sweden prohibits us from making individual level data publicly available due to ethical concerns about identification. Researchers who are interested in replicating our work can apply for data from the Swedish Twin Registry: https://ki.se/en/research/the‐swedish‐twin‐registry.
