# Supplementary material for: The genetics of gaming: A longitudinal twin study
Source: JCPP Adv. 2023 May 28;3(4):e12179. doi: 10.1002/jcv2.12179 (PMC10694538; doi:10.1002/jcv2.12179)
Supplement: Supplementary file 1 — Supporting Information S1 [file JCV2-3-e12179-s001.docx]

**Supporting Information**

**Table S1.** Numbers and proportion of gaming frequency. (Same information as shown in Figure 1.)

|  | Age 9 | Age 15 | Age 18 |
| --- | --- | --- | --- |
| **Boys** |  |  |  |
| Almost daily | 4,222 (38.4%) | 3,531 (54.6%) | 2,474 (52.7%) |
| 3-6 times per week | 3,975 (36.2%) | 1,666 (25.8%) | 893 (19.0%) |
| 1-2 times per week | 2,308 (21.0%) | 697 (10.8%) | 590 (12.6%) |
| Once a month | 277 (2.5%) | 416 (6.4%) | 472 (10.0%) |
| Never | 205 (1.9%) | 154 (2.4%) | 268 (5.7%) |
| **Girls** |  |  |  |
| Almost daily | 2,507 (23.0%) | 536 (7.7%) | 354 (7.0%) |
| 3-6 times per week | 3,019 (27.7%) | 659 (9.5%) | 281 (5.5%) |
| 1-2 times per week | 3,416 (31.3%) | 1,065 (15.4%) | 509 (10.0%) |
| Once a month | 992 (9.1%) | 2,266 (32.7%) | 1,323 (26.1%) |
| Never | 970 (8.9%) | 2,412 (34.8%) | 2,603 (51.3%) |

**Table S2.** Polychoric correlations of gaming, adjusted for birth year^a^

|  |  |  | Polychoric correlations | |
| --- | --- | --- | --- | --- |
|  |  |  | Age 15 | Age 18 |
| Boys | Age 9 | Estimate | 0.26 (0.24-0.29) | 0.25 (0.18-0.32) |
|  |  | Sample size^b^ | 2,896 | 1,352 |
|  | Age 15 | Estimate | - | 0.57 (0.53-0.61) |
|  |  | Sample size^b^ | - | 3,040 |
| Girls | Age 9 | Estimate | 0.22 (0.18-0.26) | 0.23 (0.16-0.30) |
|  |  | Sample size^b^ | 3,194 | 1,449 |
|  | Age 15 | Estimate | - | 0.51 (0.48-0.55) |
|  |  | Sample size^b^ | - | 3,319 |

^a^Adjusted for continuous measure of birth year and birth year squared, adjustments were made in a structural equation model.

^b^ Sample size with non-missing values at both ages.

**Table S3.** Observed intraclass correlations, adjusted for birth year.

|  | Age 9 | Age 15 | Age 18 |
| --- | --- | --- | --- |
| MZ female | 0.90 (0.89-0.91) | 0.85 (0.83-0.87) | 0.84 (0.82-0.87) |
| MZ male | 0.89 (0.87-0.90) | 0.89 (0.88-0.91) | 0.84 (0.81-0.87) |
| DZ female | 0.80 (0.78-0.82) | 0.73 (0.70-0.76) | 0.72 (0.68-0.76) |
| DZ male | 0.73 (0.70-0.75) | 0.58 (0.53-0.63) | 0.57 (0.51-0.63) |
| DZ opposite sex | 0.52 (0.50-0.55) | 0.25 (0.20-0.30) | 0.27 (0.20-0.33) |

**Table S4.** Estimated univariate ACE, adjusted for birth year.

|  | Age 9 | Age 15 | Age 18 |
| --- | --- | --- | --- |
| A female | 20.2% (16.2-24.3) | 23.3% (16.7-30.0) | 25.1% (15.6-34.6) |
| A male | 32.0% (26.7-37.3) | 63.1% (52.6-73.7) | 53.9% (41.1-66.7) |
| C female | 69.7% (65.9-73.4) | 61.8% (55.7-67.8) | 59.4% (50.7-68.1) |
| C male | 56.7% (51.7-61.6) | 26.3% (16.0-36.6) | 30.2% (18.3-42.2) |
| E female | 10.1% (09.1-11.1) | 14.9% (13.1-16.7) | 15.6% (13.0-18.1) |
| E male | 11.3% (10.0-12.6) | 10.6% (8.8-12.3) | 15.9% (13.0-18.8) |
| r_fm | -0.818 (-1.225--0.412) | -0.789 (-1.370--0.208) | -0.853 (-1.576--0.130) |

Note: ‘r_fm’ are estimated genetic correlations between males and females. Negative values are not readily interpretable.
